# The Role of Lutein in Eye-Related Disease

**DOI:** 10.3390/nu5051823

**Published:** 2013-05-22

**Authors:** Keyvan Koushan, Raluca Rusovici, Wenhua Li, Lee R. Ferguson, Kakarla V. Chalam

**Affiliations:** Department of Ophthalmology, University of Florida, 580 West 8th Street, Tower II, 3rd Fl., Jacksonville, FL 32209, USA; E-Mails: Keyvan.Koushan@jax.ufl.edu (K.K.); Raluca.Rusovici@jax.ufl.edu (R.R.); wenhua_li2003@yahoo.com (W.L.); Lee.Ferguson@jax.ufl.edu (L.R.F.)

**Keywords:** lutein, age-related macular degeneration, macular pigment, heterochromatic flicker, cataract, macular diseases, uveitis, retinitis pigmentosa

## Abstract

The lens and retina of the human eye are exposed constantly to light and oxygen. *In situ* phototransduction and oxidative phosphorylation within photoreceptors produces a high level of phototoxic and oxidative related stress. Within the eye, the carotenoids lutein and zeaxanthin are present in high concentrations in contrast to other human tissues. We discuss the role of lutein and zeaxanthin in ameliorating light and oxygen damage, and preventing age-related cellular and tissue deterioration in the eye. Epidemiologic research shows an inverse association between levels of lutein and zeaxanthin in eye tissues and age related degenerative diseases such as macular degeneration (AMD) and cataracts. We examine the role of these carotenoids as blockers of blue-light damage and quenchers of oxygen free radicals. This article provides a review of possible mechanisms of lutein action at a cellular and molecular level. Our review offers insight into current clinical trials and experimental animal studies involving lutein, and possible role of nutritional intervention in common ocular diseases that cause blindness.

## 1. Introduction

Lutein belongs to the xanthophyll family of carotenoids, which are synthesized within dark green leafy plants, such as spinach and kale [1,2]. On average, Americans consume a daily intake of 1.7 mg lutein [3]. The purified crystalline form of lutein has been generally recognized as being safe for supplementation into foods and beverages [4]. Lutein is absorbed with fat in the gastrointestinal system and transported via lipoproteins. Apolipoprotein E is involved in the transport of lutein in serum [5], with transference being facilitated primarily by High Density Lipoproteins (HDLs) (52%) and Low Density Lipoproteins (LDLs) (22%) [6] In the presence of cholesterol, lutein segregates out from saturated lipid regions (liquid-ordered phase) on cell membranes and accumulate into unsaturated phospholipids in order to form carotenoid-rich domains [7] Tissue specific lutein concentrations are dependent on dietary intake [8,9]. Following dietary ingestion, lutein concentrations of approximately 0.2 μM circulates throughout the body to target tissue sites such as the retina where they are incorporated and serve a functional role in maintenance of tissue homeostasis [3].

In the primate eye lutein, along with zeaxanthin and its isomer meso-zeaxanthin, represent the primary pigment molecule distributed within the macula [6,10]. Lutein and its stereoisomer zeaxanthin are distinguished from other carotenoid compounds based on the chemical composition of hydroxyl group attachments to their structures [11,12]. These macular pigment compounds, which are responsible for the yellow hue of the macula lutea are concentrated in the outer and inner plexiform layers [13,14] as well as in rod outer segment within the macula [15]. Lutein varies in its distribution in the eye. It is found in higher amounts within the peripheral retina, RPE, choroid and ciliary body, while demonstrating small concentrations in the iris and lens [16,17]. Dietary concentrations between 6 and 20 mg per day of lutein have been associated with a reduced risk of ocular disorders such as cataracts and age-related macular degeneration [18,19]. The effects of lutein and other antioxidants in mitigating early onset age related ocular and neurological diseases have been well documented [20,21,22,23]. Macular pigments such as lutein have biochemical significance to ocular health by averting disease onset as well as sustaining visual functionality.

## 2. Structure and Biochemistry of Lutein

### 2.1. Lutein in the Retina

**Figure 1:**
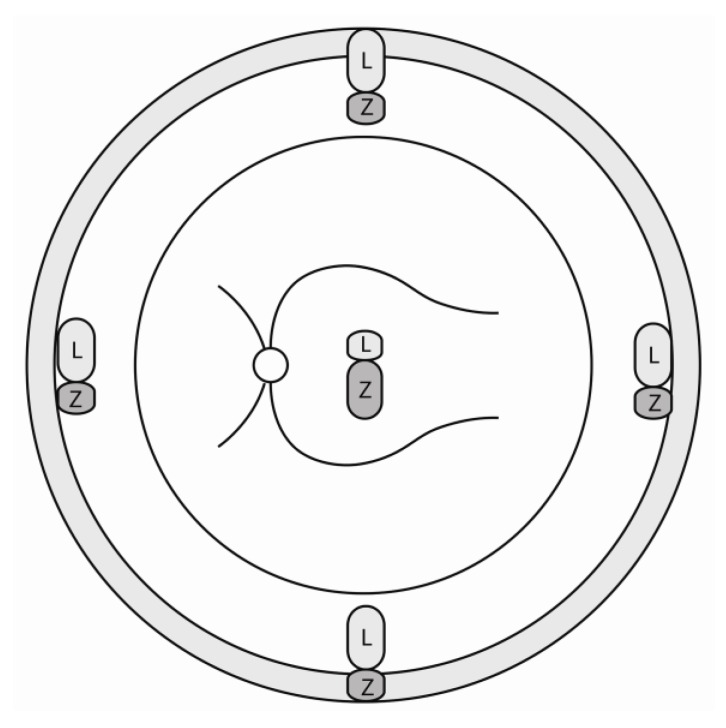
Schematic representation of the ratio of lutein to zeaxanthin in central and peripheral retina; L: lutein; Z: zeaxanthin.

In terms of tissue concentrations, the presence of lutein and zeaxanthin in the retina represent the highest seen among any other human tissue type. Despite this apparent abundance, the distribution of these carotenoids in the retina is not uniform. In the human fovea, lutein is found in lower quantities relative to zeaxanthin by a ratio of approximate 1:2 [24,25]. In general, the relative amounts of the retinal carotenoids decrease as the point of reference travels farther away from the fovea. This ultimately leads to a ratio amount of 2:1 between lutein and zeaxanthin in the peripheral retina (Figure 1). Moreover, the overall macular pigment optical density for both carotenoids decreases 100 fold in the periphery in comparison to the foveal region [26,27,28,29,30].

Macular pigment density represents an indirect measure of the amount of lutein present in the body [31,32,33]. There are several methodologies available that provide some level of quantification of macular pigment density in the retina. Analytical techniques such as heterochromatic flicker [34], fundus reflectometry [35], lipofuscin autofluorescencespectrometry, Resonance Raman Spectroscopy [36], and high performance liquid chromatography (HPLC) [37] provide methods to determine optical density of lutein and other macular pigments. A fairly novel methodology, *in vivo* snapshot hyperspectral imaging with non-negative matrix factorization, analyzes hyperspectral signatures from macular pigments as a means to distinguish biochemical components unique to individual pigment molecules [38]. 

### 2.2. Role of Lutein in Prevention of Hypoxia-Induced Cell Damage in the Eye

**Figure 2:**
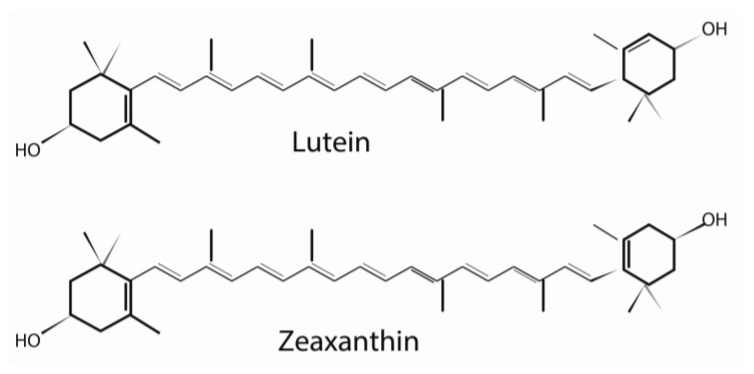
Chemical structures of lutein and zeaxanthin.

**Figure 3:**
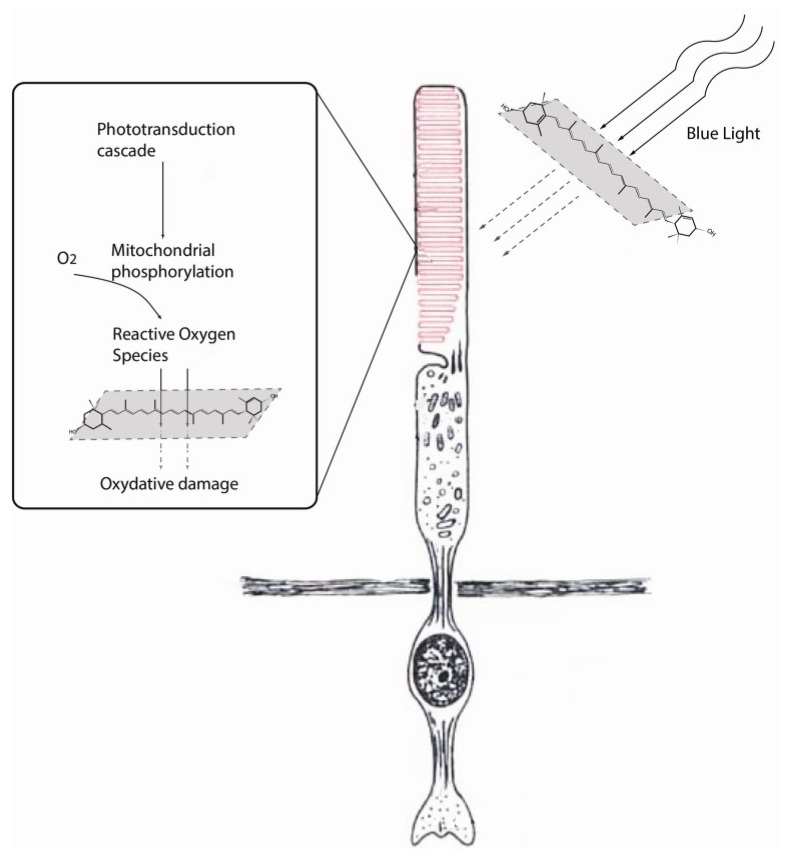
Proposed mechanisms for the protective role of lutein against cellular damage. Lutein reduces the amount of blue light that reaches the photoreceptors. In addition, lutein directly scavenges the reactive oxygen species, thereby preventing them from damaging DNA and protein molecules.

Free radicals, as defined by the presence of one or more unpaired electrons in an atom or groups of atoms, come in many different forms. The most common type of free radical in biological system is the reactive oxygen species (ROS). ROS such as singlet oxygen species are generated in the retina because of oxygen consumption as well as high energy photon light conversion into electrochemical signaling [39,40,41,42,43,44]. ROS generated in the retina are believed to be the byproducts of extramitochondrial oxidative phosphorylation in the rod outer segment [44,45]. The accumulation of oxygen radicals and lipid peroxidation, resulting from increased retinal oxygen utilization, has been postulated as a mechanism for photoreceptor apoptosis. Oxidative damage occurring in the rod outer segment causes the release of extra-mitochondrial components like cytochrome c, which initiates apoptosis via caspase 9 activation. One of the major protective roles lutein has in the retina is to serve as an oxygen free radical scavenger during oxidative stress conditions. The ability of lutein to provide effective removal of free radicals, such as singlet oxygen particles, is primarily governed by the chemical structure of two hydroxyl groups acting as strong sinks for reactive oxygen species (Figure 2) [46,47,48]. Xanthophylls and carotenes are considered the most efficient singlet oxygen scavengers among the carotenoid families [49,50]. The difference in the singlet oxygen extinguishing efficiency primarily relates to the number of double bonds present in the carotenoids. Carotenoids with greater amounts of double bonds are better able to lower their triplet energy state. As a result, molecules such as lycopene, β-carotene, zeaxanthin, cryptoxanthin, and α-carotene can achieve triplet states that facilitate energy transfer with singlet oxygen species. The effective dose (ED50) value of lutein as a free radical scavenger is 0.7 μM. In addition, lutein selectively absorbs blue light, due to its peak absorption spectrum of 446 nm. Blue light produces more light induced damage (100-fold) than orange light, depending on the exposure time. As a consequence of its filtering capabilities, lutein is effective in preventing photoreceptor damage produced by blue light (Figure 3) [51].

Several *in vivo* and *in vitro* studies have investigated the pharmacokinetic properties of lutein and zeaxanthin as well as the effects xanthophyll supplementation has on prevention of cellular damage due to photochemical and oxidative stress. Snodderly demonstrated that supplementation of cynomolgus and squirrel monkeys with lutein and zeaxanthin resulted in an increase in the plasma concentration of carotenoids [52]. Another study showed serum levels of zeaxanthin in rhesus monkeys can be raised by supplementation with the carotenoids from an extract of Fructus lycii [53]. Some studies have demonstrated reversal of lifelong absence of xanthophylls in primates with lutein supplementation, and subsequent amelioration of acute blue-light photochemical damage [39,54,55,56]. In retinal pigment epithelial cells, fed native or UV-irradiated photoreceptor outer segments and cultured in 40% oxygen, lutein significantly reduced lipofuscin formation [57,58]. In RGC-5 rat ganglion cell lines, lutein treatment prevented cellular death following oxidative damage via H_2_O_2_. Similar results were obtained in immortalized Müller cells [59,60].

## 3. Role of Lutein in Age-Related Macular Degeneration (AMD)

The macula lutea is located in the central and posterior portion of the retina and possesses the highest concentration of photoreceptors, which are responsible for central vision and high-resolution visual acuity. It is a circular area 5–6 mm in diameter that possesses a characteristic yellow pigment that is made up entirely of lutein and zeaxanthin.

Light-induced retinal damage depends largely on the wavelength, exposure time, and intensity of light. For instance, the blue light (440 nm) requires 100 times less intensity to cause damage than orange light (590 nm). The presence of carotenoids in the macula capable of absorbing light of the blue range wavelength would indicate that they serve a protective function. Specifically lutein appears to play a specific role as a photoprotective agent, effectively screening out the damaging blue light from causing excessive damage on the photoreceptors.

AMD is a degenerative process of the macula and is the principle cause of blindness among people age 65 and older in Western countries. AMD can be classified into two categories: non-exudative (or dry) and exudative (or wet) AMD. The former is characterized by accumulation of soft drusen caused by photo-oxidative damage and de-pigmentation of the retinal pigment epithelium. The latter is characterized by neovascularization of the macula, and accumulation of scar tissue.

AMD is a multifactorial disease. Among the important risk factors for AMD are age, genetic susceptibility, sunlight exposure, cigarette smoking, and poor nutritional status.

Combined with the fact that lutein and zeaxanthin are the only carotenoids found in the macula and comprise the macular pigment, this suggests that the observed protective effects of high fruit and vegetable intake may be due primarily to lutein and zeaxanthin intake.

### 3.1. Experimental Studies

The mechanism by which lutein is transported from blood to RPE and photoreceptors is not well known. It is shown, however, that when lutein and other anti-oxidants (zeaxanthin, lycopene, or α-tocopherol) are added to rabbit and bovine RPE cells under normobaric hyperoxia, formation of lipofuscin is significantly reduced in these cells [61].

A large case-control genetic study on French and North American patients with and without AMD analyzed single nucleotide polymorphism (SNP) rs5888 of the SCARB1 gene, coding for SRBI, which is involved in the lipid and lutein pathways. To investigate whether this SNP polymorphism has an association with AMD, the investigators identified AMD and control subjects who did not carry two known genetic variations associated with AMD (ARMS2 and CHF). They showed that in French and pooled populations (French and North American) without these known mutations, there is a strong association between AMD and SCARB1 gene. Additionally, Subgroup analysis in exudative forms of AMD revealed a pooled OR of 3.6 for individuals heterozygous for rs5888 [62].

Laser-induced choroidal neovascularization (CNV) is widely used in animal models for studying diseases that are associated with CNV, including Age-related Macular Degeneration (AMD). Choroidal vessels invade the subretinal space after photocoagulation in mice. In one study lutein was administered with 0.1% di-methyl sulfoxide (DMSO) as a vehicle daily for 3 days (1, 10, or 100 mg/kg body weight). Lutein significantly inhibited IFK-β-degradation at 4 h in the murine RPE-choroid complex in a dose-dependent fashion. In contrast, lutein administration in mice for 4 days did not affect IFK-β levels in the RPE-choroid. Mice treated with DHMEQ, an inhibitor of NFK-β nuclear translocation, at the dose of 0.5, 1, or 5 mg/kg showed a significant and dose-dependent decrease in the index of CNV volume compared with vehicle-treated mice [63].

### 3.2. Epidemiological and Clinical Studies

In the late 1980s, a cross-sectional sample from the National Health and Nutritional Examination Survey (NHANES) was used to show that diets high in fruits and vegetables were inversely associated with AMD [64]. Such diets are also high in many carotenoids, including lutein, suggesting that dietary carotenoids may play a role in reducing the risk of AMD [65].

The first epidemiological study to show a direct relationship between lutein intake and AMD risk was reported by Seddon *et al.* in 1994 [18]. Among the specific carotenoids, lutein and zeaxanthin were most strongly associated with decreased AMD risk (57% lower risk for highest quintile of lutein intake). Consistent with this finding was the inverse association between intake of spinach and collard greens, two foods richest in lutein and zeaxanthin, and AMD risk. This was the first study to indicate that individuals deficient in lutein intake may be at higher risk for AMD.

In 1992, the Eye Disease Case-Control Study Group showed that total serum carotenoids (lutein, zeaxanthin, β-carotene, α-carotene, cryptoxanthin and lycopene) were inversely related to AMD risk [66]. Further analysis showed that prevalence of AMD among those in this sample with highest total serum carotenoid concentration was 66% lower than those with the lowest levels. These studies helped provide a basis for the hypothesis that dietary carotenoids may have a protective effect against AMD.

Other observational studies have demonstrated a relationship between lutein and other related outcomes of eye health. A group from the University of New Hampshire examined the relation between serum lutein, lutein intake and macular pigment density (MPD) in a group of 278 healthy volunteers [67]. Their analysis revealed that both serum lutein and dietary lutein significantly correlate with MPD in a positive manner-the higher the level in serum or in the diet, the higher the MPD. These results are consistent with previous observational studies showing a protective effect of serum and dietary lutein against AMD [18,66], and suggest that dietary sources of lutein can influence the amount of lutein deposited in eye tissues.

A revolutionary technique known as Resonance Raman Spectroscopy was used in another observational study to correlate the use of lutein supplements with Macular Pigment Density (MPD) in AMD patients [68]. In a group of age-matched AMD patients and controls, the average MPD was significantly lower in AMD patients compared to controls as long as the subjects were not consuming high-dose lutein supplements. MPD, however, was significantly higher (in the normal range) in AMD patients consuming a lutein supplement (≥4 mg per day) relative to those not receiving the supplement. This study showed that lutein supplementation is associated with increased macular pigmentation and suggests that supplementation may contribute to maintaining eye health.

In another study on enucleated eyes, the investigators obtained donor eyes from AMD patients and control subjects, and measured the actual concentrations of lutein and zeaxanthin in the central regions of the retina (area including and surrounding the macula). Within the inner region (area most closely surrounding the macula), those subjects possessing the highest concentration of lutein were 82% less likely to have AMD relative to those with the lowest concentration [27].

A placebo-controlled, double-masked parallel group study (LISA: Lutein Intervention Study Austria), 126 patients with AMD were randomized into two groups: supplementation with Lutein or placebo for 6 months. Lutein supplementation was found to significantly increase MPOD (Macular Pigment Optical Density), but it showed no effect on macular function (as assessed by microperimetry) or visual acuity [69]. 

Lutein supplementation has also been shown to improve multifocal electroretingram (mfERG) response in AMD patients. Ma *et al.* have recently randomized 108 AMD patients to receive 10 mg/day lutein (*n* = 27), 20 mg/day lutein (*n* = 27), 10 mg/day lutein plus 10 mg/day zeaxanthin (*n* = 27), or placebo (*n* = 27) for 48 weeks. Their results showed that mfERM responses significantly improved for the 20 mg lutein group and for the lutein and zeaxanthin group. They also showed that macular pigment optical density also improved in all treatment groups [19,21].

Age-Related Eye Disease Study 2 (AREDS2) is a randomized, placebo-controlled, multicenter study designed to determine whether supplementation with 10 mg of lutein and 2 mg of zeaxanthin per day can slow the rate of progression of age-related macular degeneration (AMD) in persons aged 50 to 85 with bilateral intermediate AMD or advanced AMD in 1 eye. All subjects are randomly assigned to placebo (*n* = 1012), Lutein/zeaxanthin (10 mg/2 mg; *n* = 1044), ω-3 long-chain polyunsaturated fatty acids (LCPUFAs; *n* = 1069), or the combination of Lutein/zeaxanthin and ω-3 LCPUFAs (*n* = 1078). All participants are also offered a secondary randomization to 1 of 4 variations of the original AREDS formulations [70]. The results of this study are not published yet. One of the later reports of the original AREDS study, however, has showed that higher dietary intake of lutein/zeaxanthin was independently associated with reduced likelihood of having neovascular AMD, geographic atrophy, and large or extensive intermediate drusen [71]. 

## 4. Lutein Role in Diabetic Retinopathy

### 4.1. Experimental Studies

Diabetic retinopathy in animal models can be induced using streptozocin, a compound that destroys pancreatic insulin-producing β cells. Mice with diabetes show a significant decrease in body weight and a significant increase in blood glucose. As a consequence, retinal ganglion cells and amacrine cells in the inner nuclear layer (INL) undergo apoptosis in this animal model. Results show that lutein prevents reactive oxygen species formation in these diabetic mice and rats. Reactive oxygen species (ROS) in the retina were measured using dihydroethidium and visual function was evaluated by electroretinograms. The decreased amplitude of the oscillatory potentials in the diabetic mice was reversed by administration of lutein [72,73].

### 4.2. Epidemiological and Clinical Studies

The role of lutein in diabetic retinopathy has not been well studied in human subjects. Only one prospective study on patients with non-proliferative diabetic retinopathy by Hu *et al.* showed that the serum concentration of lutein and zeaxanthin is significantly lower in these patients compared to normal subjects. Their results also suggest that lutein and zeaxanthin supplementation in these patients lead to improvement of visual acuity and decrease in foveal thickness [73]. Their study suggests that lutein and zeaxanthin supplementation may potentially be used as therapeutic agents in treating non-proliferative diabetic retinopathy.

## 5. Lutein in Retinal Detachment

### 5.1. Experimental Studies

Retinal detachment was established by subretinal injections of 1.4% sodium hyaluronate in Sprague-Dawley rats. Retinal detachment is traditionally associated with severe photoreceptor cell death. When lutein was given shortly after induction of retinal detachment, it prevented apoptsis of the cells in the outer nuclear layer [74].

### 5.2. Clinical Studies

Only one study has found high levels of lutein and retinol in the subretinal fluid of a retinal detachment patient. The study found very little β-carotene in subretinal fluid. Lutein was the major carotenoid peak in subretinal fluid (41.4 ± 14.1 ng/mL). The authors suggested that the high proportion of lutein and very low amount of β-carotene in the subretinal fluid support the occurrence of a highly selective transport mechanism of lutein from the blood to the retina [75].

### 5.3. Lutein in the Lens

Lutein and zeaxanthin are the only carotenoids present in the crystalline lens [76,77]. Cataract is the opacification of the crystalline lens and is caused by precipitation of lens proteins. The development of cataract is facilitated by oxidative damage and often results in impaired vision or blindness.

### 5.4. Experimental Studies

Lutein reduces fullerol-mediated phototoxic damage of the lens proteins and DNA in human lens epithelial cells in cell culture models. This protective effect is likely due to lutein’s antioxidant properties. Other natural antioxidants (*N*-acetyl-l-cysteine nor l-ascorbic acid), however, did not provide any protection for human lens epithelial cells [78]. 

Animal studies have shown that lutein treatment slows the development and progression of cataracts in diabetic rats (more rats––10 out of 16––presented with clear lenses if treated with lutein *vs.* the non-treated diabetic group). Lipid peroxidation is significantly increased in diabetic lens (up to three-fold) and is reduced by lutein administration in this rat model [3,26,79,80]. In human lens cells, lutein supplementation increases GSH levels which protect against oxidative stress.

### 5.5. Epidemiological and Clinical Studies

The Carotenoids in Age-Related Eye Disease Study, an ancillary study of the Women’s Health Initiative Study showed that women in the highest quintile category of diet or serum levels of lutein and zeaxanthin were 32% less likely to have nuclear cataracts as compared with those in the lowest quintile category [81]. A cross-sectional study of 376 subjects found an inverse relationship between lens optical density (LOD) and MPOD, suggesting that lutein and zeaxanthin may retard aging of the lens [82]. An ancillary study of the Nurses’ Health Study cohort on the effect of nutrition on development of age-related cataracts found that the prevalence of nuclear opacification was significantly lower in the highest nutrient intake quintile category relative to the lowest quintile category for vitamin C, vitamin E, riboflavin, folate, beta-carotene, and lutein/zeaxanthin. However, after adjustment for other nutrients, only vitamin C intake remained significantly associated (*p* = 0.003) with the prevalence of nuclear opacities. The prevalence of nuclear cataracts was significantly lower (*p* < 0.001) in the highest vitamin C intake quintile category relative to the lowest quintile category [83].

## 6. Lutein and the Uvea

Uveitis, a common ophthalmic disorder, is responsible for approximately 10% of blindness in western countries [84,85]. It may be caused by autoimmune disorders, infections or exposure to toxins. 

### 6.1. Experimental Studies

Reactive oxygen species (ROS) play an important role in mediating the inflammatory signals induced by lipopolysaccharides (LPS). It is suggested that natural antioxidants exert protective effects on the LPS-induced uveitis [86].

In an LPS-induced uveitis mice model, oral administration of lutein (125 and 500 mg/kg/day for five days) reduced the nitric oxide level in eye tissues [87]. The same study showed that lutein decreased the malondialdehyde content, increased the oxygen radical absorbance capacity level, glutathione, the vitamin C contents and total superoxide dismutase (SOD) and glutathione peroxidase (GPx) activities and further increased expressions of copper-zinc SOD, manganese SOD and GPx mRNA. Hence the antioxidant properties of lutein contributed to the protection against LPS-induced uveitis, partially through interfering with the inflammatory process.

Multiple animal studies on the neuroprotective effects of lutein against retinal neural damage caused by inflammation in endotoxin-induced uveitis (EIU) [59,88] have shown that the lutein has a dose-dependent anti-inflammatory effect on EIU. The possible mechanism for this effect of lutein may depend on its ability to inhibit the activation of NF-κB and the subsequent inhibition of pro-inflammatory mediators.

### 6.2. Epidemiological and Clinical Studies

Clinical administration of lutein for prevention or treatment of diseases of the uvea (including uveitis and CNV) has not been reported. 

## 7. Lutein Supplementation

Since lutein is completely insoluble in water, its incorporation into carrier systems has been studied to optimize its nutritional delivery method. Traditionally, lutein is dissolved in an organic solvent and nano-assemblies are obtained by emulsion–solvent evaporation, associating lutein with the amphiphilic cyclodextrin C4:7 at 1:6 molar ratio in aqueous medium. The nano-assemblies allow increased carotenoid solubility in water compared to carotenoid by itself.

It has been suggested that 6 mg of lutein per day, either through diet or using supplements is likely effective in reducing the risk of cataracts and AMD. Although the optimal dose for lutein supplementation has not been established yet, the most common dose in commercial products is 10 mg/day. Lutein is found in many natural products including broccoli, spinach, kale, corn, orange pepper, kiwi fruit, grapes, orange juice, zucchini, and squash. There is 44 mg of lutein per cup of cooked kale, 26 mg/cup of cooked spinach, and 3 mg/cup of broccoli [89]. Toxicity of dietary intake of lutein (supplemental 35 mg per day) has been studied in rats and results show no serious adverse effects [90]. 

## 8. Discussion and Conclusions

There are several epidemiological studies that link lutein supplementation with decreased risk of AMD. Lutein supplementation has also been positively linked to increased macular pigment density and improved multifocal electroretingram responses. Building on the findings of the original Age-Related Eye Disease Study, the AREDS2 study is further investigating whether supplementation with lutein and zeaxanthin, in addition to original AREDS formula, would add additional benefit in improving AMD outcomes. Lutein has a significant role in protecting against AMD, most likely through its absorption of the harmful blue light, as well as its inherent antioxidant properties.

The role of lutein in protecting against diabetic retinopathy is not as well established as it is the case for AMD. Serum levels of lutein has been shown to be lower in NPDR patients and lutein supplementation has been shown to improve NPDR [73]. The antioxidant properties of lutein would likely explain its protective function in diabetic retinopathy. Further epidemiologic studies are needed to establish a stronger link between lutein and improvement of diabetic retinopathy.

Several lines of evidence have also suggested a protective role for lutein in the development of nuclear sclerosis cataracts. Considering that oxidative damage of lens proteins plays a major role in development of cataracts, anti-oxidative functions of lutein explain its likely role in slowing the formation of cataracts.

There are weaker lines of evidence to suggest protective roles for lutein against uveitis, and its possible role in the pathogenesis of retinal detachment. Further studies are needed to clarify whether lutein has a role in these ocular diseases.

In conclusion, the antioxidant, anti-inflammatory and blue light-absorptive properties of lutein provide its many protective roles in various ocular diseases especially AMD and cataracts. Lutein has become known as the “eye vitamin” and its dietary intake is important in maintaining its concentration in human lens and retina.
